# A comparison of the response of diploid and triploid Atlantic salmon (*Salmo salar*) siblings to a commercial furunculosis vaccine and subsequent experimental infection with *Aeromonas salmonicida*

**DOI:** 10.1016/j.fsi.2016.08.049

**Published:** 2016-10

**Authors:** Lynn Chalmers, Kim D. Thompson, John F. Taylor, Sean Black, Herve Migaud, Ben North, Alexandra Adams

**Affiliations:** a Institute of Aquaculture, University of Stirling, Stirling, FK9 4LA, UK; b Moredun Research Institute, Pentlands Science Park, Bush Loan, Midlothian, EH26 0PZ, UK; c Europharma Scotland Ltd., Unit 5 Dunrobin Court, 14 North Avenue, Clydebank Business Park, G81 2QP, UK; d PHARMAQ Ltd., Unit 15 Sandleheath Industrial Estate, Fordingbridge, SP6 1PA, UK

**Keywords:** Triploid, Furunculosis, Challenge, Vaccination, Adhesion, Immune response

## Abstract

Sterile triploid fish represent a solution to the problems associated with sexual maturation and escapees in aquaculture. However, as disease outbreaks continue to cause significant economic losses to the industry, it is essential that the response of triploids to disease and disease treatments be characterised. The aim of this study was to compare the response of triploid Atlantic salmon to a commercial furunculosis vaccine with that of diploid fish, and to assess the vaccine efficacy in the two ploidies through an experimental infection with *Aeromonas salmonicida*. Diploid and triploid Atlantic salmon were injected intraperitoneally with either phosphate buffered saline, liquid paraffin adjuvant or a commercial furunculosis vaccine. Following vaccination, growth, adhesion scores and a variety of assays to assess immune function, such as respiratory burst and antibody response, were measured. Vaccination did not have a significant effect on the weight of either ploidy prior to challenge at 750° days. Adhesion scores were significantly higher in vaccinated fish compared to unvaccinated fish, although no effect of ploidy was observed. Ploidy significantly affected respiratory burst activity following vaccination, however, with triploids exhibiting higher activity than diploids. Combined with lower white blood cell numbers observed in the triploids, it may be that this low cell number is compensated for by increased cellular activity. Ploidy however, did not have a significant effect on complement activity or antibody response, with significantly higher antibody levels detected in all vaccinated fish compared to unvaccinated controls. In addition, both ploidy groups were well protected following challenge with no difference in the relative percentage survival. Based on these results, it appears that ploidy does not affect the severity of adhesions that result post-vaccinate or in the fish's immune response following vaccination, and the furunculosis vaccine performs equally well in both diploid and triploid Atlantic salmon.

## Highlights

• Commercial furunculosis vaccine effective in protecting triploid Atlantic salmon.
• Ploidy did not affect the severity of the adhesions occurring in vaccinated fish.
• Differences in immune response may due to compensatory mechanism in triploids.

## 1 Introduction

In recent years, the Atlantic salmon (*Salmo salar*) industry has paid particular attention to the issues associated with sexual maturation prior to harvest and issues related to escapees [1], [2], [3], [4]. Sexual maturation in fish causes the transfer of energy from normal somatic growth to gonadal development. This is known to have adverse effects on body growth rates and flesh quality, and may increase incidences of disease and mortality [5], [6], [7]. Sexually mature fish that escape from production sites also have the potential to interact with wild fish, impacting on the genetics and fitness of the wild population [8]. Triploid salmon are the only commercially available and acceptable means of achieving sterility in fish, and are increasingly being used as a method to control sexual maturation [2], [4], [9], [10].

Triploidy can be readily induced through the application of a hydrostatic or temperature ‘shock’ to newly-fertilised eggs and the process has been optimised for several commercially important species in aquaculture including Atlantic salmon, rainbow trout (*Oncorhynchus mykiss*), brown trout (*Salmo trutta*), turbot (*Scophthalmus maximus*) and grass carp (*Ctenopharyngodon idella*) [4], [11], [12], [13]. This ‘shock’ treatment prevents second meiotic division and causes the retention of the second polar body, which results in three sets of chromosomes rather than two, and in turn sterility in triploid fish [11], [12]. Importantly, only females appear to be fully sterile as males have been found to develop gonads, although sperm is aneuploid and as a result not functional [14]. However, despite the clear advantages of being sterile, if triploid Atlantic salmon are to be considered for commercial production, they must perform equally as well as diploids in all aspects of their biology and physiology.

Many studies over recent years have investigated triploid salmon physiology and performance, including effects on egg incubation [9], smoltification [15], growth [2], [7], [16], [17], [18], deformity and cataracts [17], [19]. There are very few studies that have focussed on triploid salmon health and immunity, however. Disease and resulting health issues are considered one of the largest single causes of economic losses in aquaculture and, as such, represent a significant constraint to the continued development and success of the industry [20], [21]. Understanding how triploid fish cope with health challenges is therefore crucial and it is important to characterise their robustness. Empirical evidence suggests no differences in mortality of triploid salmon compared to diploid siblings when challenged with disease in commercial settings, although few scientific assessments have been carried out. A study by Frenzl et al. [22] showed similar infection levels between ploidies when challenged with the ectoparasite sea lice, *Lepeophtheirus salmonis,* following both experimental and natural challenges. However, there is a clear lack of data on triploid salmon response to bacterial and viral challenges and conflicting results have been reported in other fish species [23], [24].

Furunculosis, caused by the bacterium *Aeromonas salmonicida* subsp. *salmonicida*, is recognised as one of the most commercially significant infectious diseases of Atlantic salmon [25], [26]. This pathogen causes a haemorrhagic septicaemia, deep ulcerative lesions and necrosis of the kidney, and can result in high levels of mortality [27]. As a result, effective commercial vaccines against furunculosis have been developed and are widely used in salmon aquaculture [28]. Application of vaccines can be stressful for the fish, and can result in side effects, compromising fish welfare. In a study by Fraser et al. [29], it was shown that vaccination with Norvax MINOVA 6 Vet (Norvax^®^, Intervet International B.V., Boxmeer, Netherlands), a multivalent vaccine against furunculosis, classical vibriosis, coldwater vibriosis, and infectious pancreatic necrosis (IPN), induced higher abdominal adhesions in triploid salmon, compared with diploid fish in out of season S0 smolts [29]. Further studies are clearly required to fully elucidate the response of triploid salmon to infectious agents and vaccination, and to determine if their responses are significantly different from their diploid counterparts.

The aim of the study was to investigate the response of triploid salmon to vaccination against furunculosis using a commercially available vaccine and assess levels of protection elicited by the vaccine through experimental infection with *Aeromonas salmonicida* compared with their diploid siblings.

## 2 Materials and methods

### 2.1 Fish stock and history

Eggs and milt were obtained from commercial Atlantic salmon broodstock stripped in October 2012 (Aquagen^®^ Atlantic QTL-innOVA^®^ IPN/PD strain Norway). Following fertilisation, half of each egg batch was subjected to a pressure shock (655 bar for 6.25 min, 37.5 min post-fertilisation at 8 °C) to induce triploidy. Eggs were then incubated at 6.0 ± 0.5 °C until eyed. Eyed eggs (21 December 2012, 372° days,°D) were supplied to the University of Stirling facilities (Howietoun Fish Farm) and incubated in complete darkness at 7.1 ± 0.3 °C until they started to hatch (7 January 2013, ∼470–500°D). First feeding commenced on 25 February 2013, (∼880°D) and temperature gradually increased (8.4 ± 1.3 °C). On 28 May 2013, fry were transferred to the Niall Bromage Freshwater Research Facility (NBFRF), Buckieburn, and maintained on constant light until late August and then simulated natural photoperiod thereafter, under an ambient water temperature (range: winter, 3 – summer, 15 °C) to produce S1+ smolts. Fish were fed a commercial diet (BioMar Inicio Plus), distributed by automatic feeders (ARVOTEK). Specific feeding rates (% tank biomass per day) were adjusted automatically according to predicted growth and daily temperature, and pellet size (0.5 mm–2.0 mm) increased with fish size. Mortality between first feeding and vaccination was 1.18% for diploids and 1.99% for triploids. On 21st January 2014, fish were transferred from the NBFRF to 0.1 m^3^ tanks (100 L; 1 L min^−1^ flow rate) at the Aquaculture Research Facility (ARF), University of Stirling, where water temperature was maintained at 9.6 ± 1.1 °C and fish were fed a 0.5% biomass diet.

### 2.2 Vaccination and sampling

Fish were vaccinated on 11th November 2013 (5 °C) using a commercial vaccination gun (Fishjector 0.1, Kaycee Veterinary Products, UK). Initial mean weight (±SEM) was 71.4 ± 2.74 g and 58.5 ± 3.57 g for diploids and triploids, respectively. Fish from both ploidy were divided into three treatment groups in triplicate tanks (60 fish tank^−1^), sedated using MS-222 (Pharmaq AS, Oslo, Norway), and then injected intraperitoneally (IP) as follows: (1) sham injected (0.02 M phosphate buffered saline, PBS group); (2) injected with adjuvant alone (liquid paraffin adjuvant, PHARMAQ AS, Oslo, Norway) (ADJ group); or (3) injected with commercial vaccine against furunculosis and infectious pancreatic necrosis (ALPHA JECT 2.2^®^ vaccine, PHARMAQ AS, Oslo, Norway) (VACC group). Following injection, fish were transferred into their designated 1 m^2^ triplicate tank (280 L; 1 L min^−1^ flow rate) for recovery. They remained on the same feeding regime, photoperiod and water temperature as previously described.

Sampling was undertaken at 5 time-points post-vaccination (50, 250, 450, 600 and 750°D) with 3 fish sampled per tank at each time-point (9 fish per treatment). Weight was assessed prior to challenge at 750°D. At all time-points, each fish was assessed for adhesion severity according to the Speilberg Scale [30]. Blood samples were taken from the caudal vein and the serum collected before being stored at −20 °C. Serum from 50 to 750°D were used to assess complement activity and antibody response, respectively. A portion of the blood sampled at 50°D was also removed for blood cell counts. At 50°D, head kidney was dissected from the fish under aseptic conditions for assessment of macrophage activity.

### 2.3 Assessment of immune parameters

All immune parameters were assessed at 50°D with the exception of antibody response which was assessed/analysed at 750°D.

#### 2.3.1 Blood cell counts

Following blood sampling, red blood cell (RBC) and white blood cell (WBC) counts were carried out according to Morgan et al. [31]. Cell counts were adjusted and expressed as cells x 10^4^ ml^−1^.

#### 2.3.2 Macrophage function

##### 2.3.2.1 Head kidney macrophage isolation

Isolation of head kidney macrophages was performed according to Secombes [32], with modifications. Head kidney was homogenised through a 100 μm nylon mesh with 5 ml L-15 medium (Sigma, UK) containing 10 μl heparin (50 mg ml^−1^) (Sigma, UK). Cell suspensions were layered onto 34%/51% Percoll gradients and centrifuged at 400 g for 30 min at 4 °C. The band of cells at the 34–51% interface was transferred into 15 ml centrifuge tubes and the volume adjusted to 15 ml with L-15 medium. Suspensions were centrifuged at 600 g for 7 min at 4 °C. The resultant cell pellets were re-suspended in 1 ml L-15 medium containing 5% foetal calf serum (Sigma, UK) and 1% potassium benzyl-penicillin/streptomycin sulphate (Sigma, UK). Cells were counted using a Neubauer haemocytometer and cell concentrations adjusted to 1 × 10^7^ cells ml^−1^.

##### 2.3.2.2 Respiratory burst

Respiratory burst activity was carried out according to Morgan et al. [31].

##### 2.3.2.3 Phagocytosis

The phagocytic activity of head kidney macrophages was assessed according to the method described by Thompson et al. [33], with modifications. Two circles were marked on glass slides using an ImmEdge pen (Vector Laboratories Inc, UK). Cell suspensions were added (100 μl circle^−1^) and slides incubated for 1 h in a humid chamber. Non-adherent cells were then removed with L-15 medium. A 0.5% (w/v) yeast (*Saccharomyces cerevisiae*) suspension was then added to one circle (100 μl) and L-15 medium (100 μl) to the other as a negative control. Slides were incubated as previously described and washed with L-15 medium. Cells were fixed for 5 min with 100% methanol (100 μl circle^−1^) and then washed five times with 70% methanol. Slides were stained with a Rapid Romanowsky staining series (TCS Biosciences Ltd, UK). Two hundred macrophages were examined by strategically scanning the slide from one side to the other using light microscopy (100x) and counted, along with the number of yeast per macrophage. Phagocytic activity, (PA), phagocytic index (PI) and phagocytic capacity (PC) were determined according to Findlay and Munday [35], using the following calculations: Phagocytic activity (PA) = (number of phagocytising macrophages/total number macrophages × 100); Phagocytic index (PI) = (total number of yeast cells consumed/number of consuming macrophages); Phagocytic capacity (PC) = (total number of macrophages containing a given number of yeast cells/total number of macrophages containing any yeast).

#### 2.3.3 Alternative complement pathway

Spontaneous haemolytic activity was determined by adapting the method described by Langston et al. [34]. Briefly, serum samples (in duplicate) were doubly diluted in 0.1% gelatine veronal buffer (GVB) (1 complement fixation tablet (Oxoid, UK), 0.1 g gelatine) in a U-well microplate (Fisher Scientific, UK) (final volume 25 μl well^−1^). A 5% (v/v) sheep red blood cell (SRBC) suspension was then added to all wells (10 μl well^−1^). Each microplate also contained control wells. As a positive control, producing 100% SRBC lysis, 0.1% (w/v) anhydrous Na_2_CO_3_ was added to wells in place of serum samples. As a negative control, eliciting 0% lysis of SRBC, 0.1% GVB replaced serum samples. All samples were incubated for 90 min at RT with constant shaking, after which the reaction was stopped by adding GVB containing 20 mM ethylenediaminetetraacetic acid (EDTA) (Sigma, UK) (140 μl well^−1^). The microplates were centrifuged at 750 g for 6 min before supernatants were transferred into flat-well microplates (Fisher Scientific, UK) (100 μl well^−1^). The absorbance was measured at 450 nm. The percentage lysis for each sample and dilution was calculated using control values. The dilution that produced 50% lysis was determined using PROBIT analysis and the reciprocal expressed as spontaneous complement haemolysis (SCH_50_%).

#### 2.3.4 Enzyme linked immunosorbent assay (ELISA)

The specific antibody response of diploid and triploid Atlantic salmon to *A. salmonicida* was measured using the methods described by Erdal and Reitan [35] and Romstad et al. [36], using sonicated antigen to coat the ELISA plates as described. Briefly, 96 well microplates (Immulon 4HBX, Fisher Scientific, UK) were coated with sonicated whole cell *Aeromonas salmonicida* MT423, diluted to 20 μg ml^−1^ in coating buffer (100 μl well^−1^). The plates were incubated overnight at 4 °C, washed with low salt wash buffer (LSWB: 0.02 M Tris, 0.38 M NaCl, 0.05% Tween 20) and post-coated for 2 h at room temperature (RT) (i.e. 20 °C) with 3% (w/v) casein (250 μl well^−1^). Doubling serum dilutions were added to the microplates along with PBS as a negative control (100 μl well^−1^) and incubated overnight at 4 °C. The microplates were then washed with high salt wash buffer (HSWB: 0.02 M Tris, 0.5 M NaCl, 0.1% Tween 20), incubating for 5 min on the last wash. Microplates were incubated for 1 h at RT with rabbit anti-trout polyclonal antibody (Aquatic Vaccine Unit, University of Stirling, Stirling, UK), diluted 1:1000 with PBS (100 μl well^−1^). After washing with HSWB, conjugate (anti-rabbit-Horseradish peroxidase, Sigma-Aldrich, USA) diluted 1:2000 with conjugate buffer (1 g bovine serum albumin (BSA) (Fisher, UK) in 100 ml LSWB), was added for 1 h (100 μl well^−1^). The reaction was developed by the addition of chromogen (3′3′5′5′-Tertamethylbenzidine dihydrochloride) in substrate buffer (0.1 M citric acid, 0.1 M sodium acetate, pH 5.4) (100 μl well^−1^) and, after 10 min, was stopped with 2 M sulphuric acid (H_2_SO_4_) (50 μl well^−1^). Absorbance was measured at 450 nm and is expressed as optical density (OD).

### 2.4 Vaccine efficacy testing

The strain of *A. salmonicida* used for the experimental infection (‘Hooke’) following vaccination was kindly provided by Dr D. A. Austin, Heriot Watt University. The challenge dose was pre-determined using three doses (6 × 10^3^, 6 × 10^2^ & 6 × 10^1^ CFU fish^−1^) with the two highest doses resulting in 100% mortality and the lowest giving 83% mortality, and the latter dose was subsequently used in the infection trial. At challenge (870°D post-vaccination, 24th March 2014), the mean weights (±SD) of diploids and triploids were 74.6 ± 13.2 g and 61.7 ± 12.5 g, respectively. Twenty fish from each triplicate ploidy/treatment tank were anaesthetised and injected IP with a 0.1 ml dose of *A. salmonicida* suspension (4 × 10^2^ CFU ml^−1^; final concentration 4 × 10^1^ CFU fish^−1^). Following injection, fish were immediately transferred into their designated 0.1 m^3^ experimental challenge tanks (100 L; 1 L min^−1^ flow rate) and allowed to recovery. As mortalities occurred, swabs from head kidney and spleen were sampled on to brain heart infusion agar (BHIA) (Oxoid, UK) plates for identification to confirm specific mortalities. Following termination of the challenge (11th April 2014), survivors were sacrificed and a random selection sampled to confirm bacterial recovery or clearance from vaccinated fish.

### 2.5 Statistical analysis

Minitab software version 16 (Minitab Inc., Pennsylvania) was used to perform basic descriptive statistics and comparisons using a significance level of 5% (P = 0.05). Prior to analysis, datasets were checked for normality using the Anderson-Darling test. Where appropriate log transformations where performed to normalise the data. Post-hoc analyses were carried out using Tukey's multiple comparison tests with values considered significantly different at *P*-values <0.05. General linear model (GLM) manipulated to three-way analysis of variance (ANOVA) was used to analyse adhesion score, with ploidy, treatment and time (sampling point) considered fixed factors. Further two-way ANOVAs were carried out for weight at 750°D, blood cell counts, respiratory burst, phagocytosis, complement activity, antibody response and challenge mortalities using only ploidy and treatment as fixed factors.

## 3 Results

### 3.1 Growth

At 750°D post vaccination, there was no significant difference in the weight of fish between the vaccinated and unvaccinated groups. For the diploid fish, their mean weight (±SD) was 79.5 ± 12.4 g, 74.7 ± 15.2 g and 69.4 ± 10.2 g for the PBS, ADJ and VACC groups, respectively. The mean weight of the triploid groups (PBS, ADJ and VACC) was 66.1 ± 12.5 g, 57.1 ± 11.5 g and 62.3 ± 12.3 g, respectively.

### 3.2 Adhesion score

**Fig. 1:**
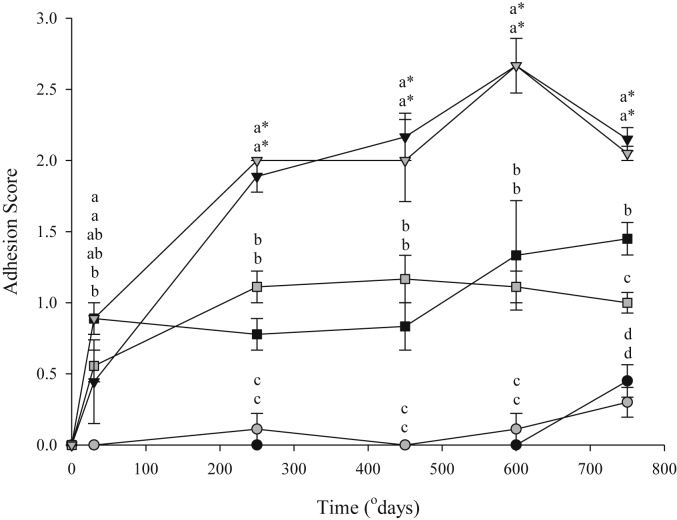
Adhesion score (mean ± SEM, n = 9) in diploid (black) and triploid (grey) Atlantic salmon injected with PBS (circles), ADJ (squares) or VACC (triangles). Significant differences between ploidy/treatments at a given time-point are indicated by different lowercase superscripts. Significant differences relative to 50°D are indicated by asterisk (*).

Ploidy did not have a significant effect on adhesion score throughout the course of the trial (Fig 1). Treatment had a significant effect on adhesion score with adhesions found in fish injected with adjuvant and vaccine, as early as 50°D. Differences between treatments (PBS, ADJ, VACC) were significant from 250°D onwards, with adhesion scores reached a mean peak value of 2.7 ± 0.2 at 600°D in the VACC groups (Fig. 1).

### 3.3 Immune response

**Fig. 2:**
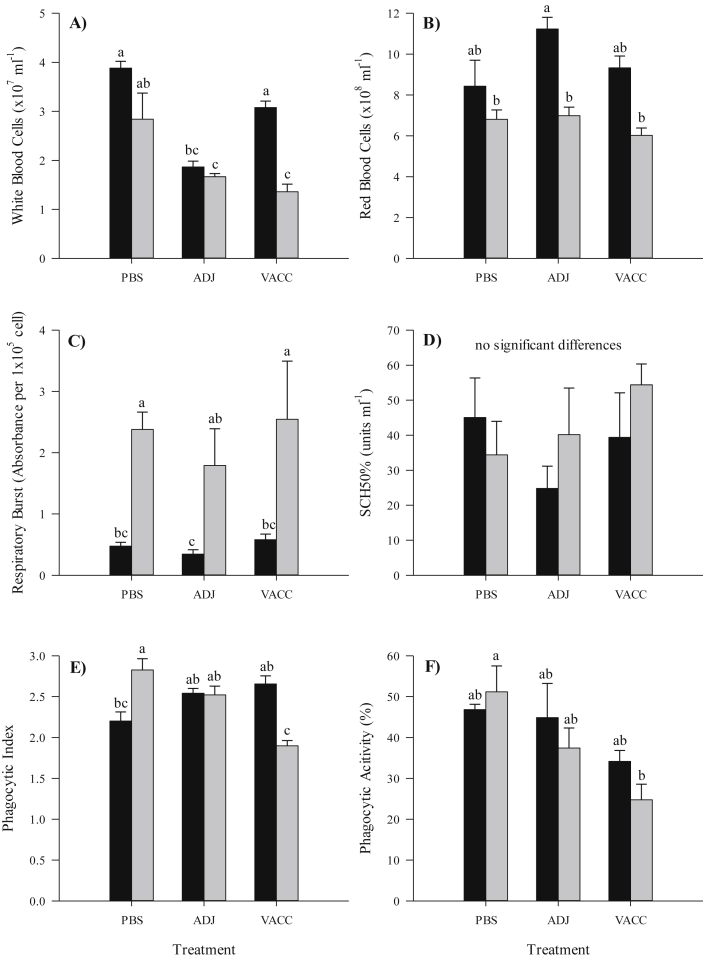
Comparison of (A) white and (B) red) blood cell counts, (C) respiratory burst activity, (D) complement activity (SCH 50%), (E) phagocytic index and (F) activity (Pa, %) between diploid (black) and triploid (grey) Atlantic salmon injected either PBS, adjuvant (ADJ) or vaccine (VACC) at 50°D. Values expressed as means ± SEM (n = 9). Significant differences are indicated by different letters.

Diploid fish had higher WBC counts than their triploid counterparts in all treatment groups, but only significantly in the VACC fish (Fig. 2A). A similar pattern was observed for RBC counts, with diploids showing significantly higher numbers than triploids in the ADJ group (Fig. 2B). For diploid fish, WBC counts in the ADJ group were significantly lower than the PBS or VACC groups, while the triploid PBS group showed significantly higher WBC counts than the other two groups. In contrast, no significant treatment differences were observed for RBC counts.

Triploid fish exhibited significantly higher respiratory burst activity by head kidney macrophages compared to diploids for all groups (Fig. 2C). Treatment did not have a significant effect on respiratory burst.

No differences in complement activity were observed between ploidy and this was not influenced by vaccination (Fig. 2D).

Macrophages of PBS injected triploid fish had a significantly higher phagocytic index (Pi) than their diploid counterparts, while the opposite was found for macrophages from the VACC groups (Fig. 2E). Within the triploid groups, the VACC group was found to have significantly lower Pi than the PBS and ADJ groups, while no significant differences were evident between the diploid groups. Ploidy was not found to have a significant effect on phagocytic activity (Pa) (Fig. 2F). Within the triploid group, the VACC group showed significantly lower Pa than the PBS group. No significant effect following vaccination was observed in the diploid fish.

Significant differences in phagocytic capacity were noted between ploidy (Table 1). In the PBS groups, diploid macrophages phagocytosed a significantly higher percentage of low yeast numbers (e.g. 1, 2), while triploid macrophages were found to consume significantly more high yeast numbers (e.g. 5, 6, 7, +). No statistical significance was noted between ploidy in the ADJ groups, but a similar pattern to that of PBS groups was observed. For the VACC groups, the opposite pattern was noted, with triploids consuming a higher percentage of low yeast numbers (1 & 2) and diploid macrophages consuming a significantly greater percentage of high yeast numbers (4, 6 & 7).

**Fig. 3:**
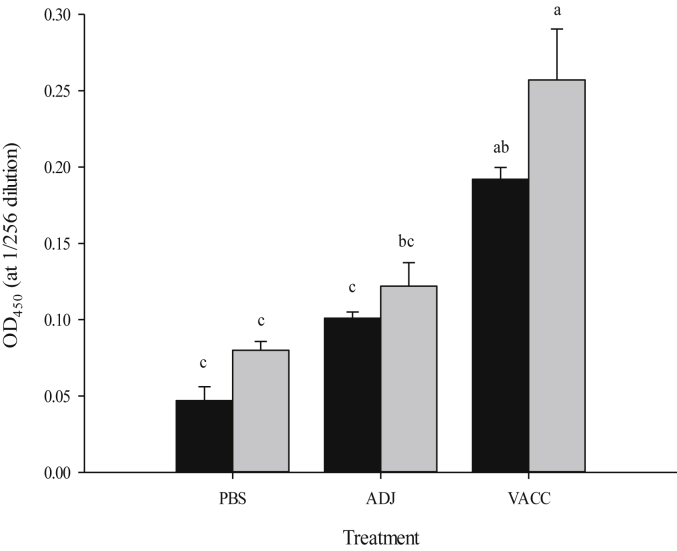
Comparison of antibody response (OD_450nm_) between diploid (black) and triploid (grey) Atlantic salmon injected either PBS, adjuvant (ADJ) or vaccine (VACC) at 750°D. Values expressed as means ± SEM (n = 9). Significant differences are indicated by different letters.

The antibody responses of all diploid and triploid groups were assessed at 750°D. There was no effect of ploidy on antibody response in any of the treatment groups examined (Fig. 3). Significantly greater antibody responses were elicited in both VACC groups compared to their respective ADJ and PBS controls.

### 3.4 Vaccine efficacy testing

**Fig. 4:**
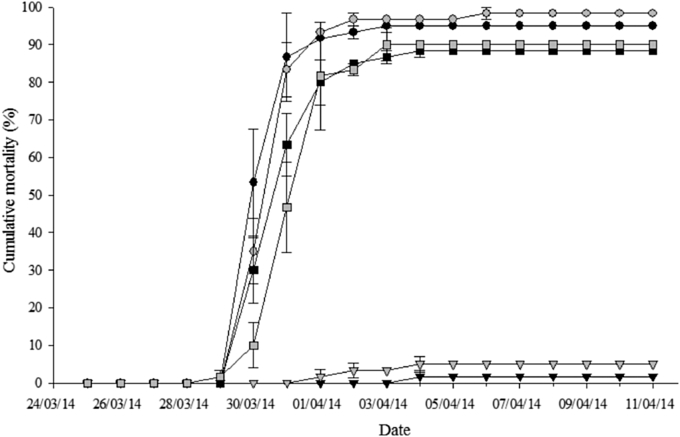
Cumulative mortality (%) of diploid (black) and triploid (grey) Atlantic salmon injected either PBS, adjuvant (ADJ) or vaccine (VACC) then experimental infected with *Aeromonas salmonicida*. Values expressed as mean treatment group mortality (%) ±SEM (n = 3). Significant differences between groups are indicated by different letters.

Following challenge with *Aeromonas salmonicida,* mortalities increased over time in both the PBS and ADJ groups while it remained low in the VACC groups (Fig. 4). Final mortality was not significantly different between ploidy for any of the treatment groups (Diploid PBS 95%, ADJ 88.3%, VACC 1.7%; Triploid PBS 98.3%, ADJ 90%, VACC 5%). Both diploid and triploid VACC groups had significantly lower mortalities compared to the PBS and ADJ groups (Fig. 4). Relative percent survival (RPS) for the diploid and triploid VACC groups was 98.3% and 94.9%, respectively. Specific mortalities were confirmed by the presence of *A. salmonicida* in swabs taken from the kidney and spleen of infected fish. *Aeromonas salmonicida* was isolated from all fish that died during the challenge and was confirmed by the appearance of dark/brown pigmented agar and by Gram stains. No *A. salmonicida* was recovered from any of the challenge survivors.

## 4 Discussion

The response of diploid and triploid Atlantic salmon siblings to vaccination with a commercial furunculosis vaccine was compared in the present study, as well as injected with either PBS or adjuvant alone, used as control groups. Innate immune responses were examined in these fish at 50°D and antibody response was assessed at 750°D, together with the level of protection elicited by the furunculosis vaccine. Side effects from vaccination e.g. growth and adhesions were examined throughout the trial as previous reports indicate poorer growth and more severe adhesions in triploids as a result of vaccination [29]. The main findings from this study indicate that triploid Atlantic salmon respond equally as well to vaccination as diploids, and ploidy does not significantly affect the severity of the adhesions that develop. Vaccine efficacy was not significantly affected by ploidy, with similar relative percent survival values obtained for both diploid (98.3%) and triploid (94.9%) fish.

The growth performance of triploids is much debated [10], [11], [15], [17], with triploid growth following vaccination relatively uncharacterised, and although vaccination did not have a significant effect on weight in triploids compared to diploids or unvaccinated fish in our study, there are reports of a reduction in weight in triploids post-vaccination from other studies [3], [29], [37]. It should be noted that our study was only performed for 4 months in freshwater, while the other studies cited lasted for considerably longer in both fresh- and seawater [3], [29]. While non-significant effects of vaccination were observed on growth in this study, supporting the use of commercial vaccines in triploid Atlantic salmon, it is recommended that vaccination should be assessed over a full production cycle.

Vaccination is known to produce other side-effects including internal abdominal adhesions [29], [38], [39]. This has serious implications at harvest, as adhesions can lead to downgrading carcass quality [40], [41]. Due to the limited assessment of the vaccination process in triploid Atlantic salmon, much is still unknown about the relationship between vaccines, their side-effects and triploids. In this study, ploidy did not have a significant impact on the severity of adhesion scores observed in diploid and triploid fish. This finding is supported by previous vaccination studies using S1+ triploid smolts, which in fact were conducted over a longer time scale [19], [29]. Vaccination did result in adhesions in both ploidies, with adjuvant alone also inducing adhesions. The PBS group of both ploidy groups consistently showed little evidence of adhesions, as expected. From 250°D onwards, adhesion scores in the VACC groups were significantly higher than both the PBS and ADJ groups, with adhesion scores in the ADJ group significantly higher than those of the PBS group. These results agree with previous studies, which found that while adjuvants alone could cause adhesions, vaccines and thus the inclusion of an antigen increased the severity of adhesion scores [30], [38], [42], [43]. Significant difference was noted in the ADJ groups between ploidies at 750°D, where triploid adhesion scores were significantly lower than that of diploid and may indicate that triploids recover more quickly from the effects of adhesions than diploids, but a longer trial would help elucidate this. From the results of this study, however, it can be suggested that triploid Atlantic salmon experience the same degree of side-effects as diploids.

Before being able to produce triploid fish commercially, it is essential that their health and immunity be carefully assessed compared to diploid counterparts [24]. Numerous studies have now been undertaken to investigate this, with reports of greater or equal disease resistance in triploids compared to diploids [3], [24], [34], [56]. As such, much remains to be elucidated about the effects of triploidy on health and immunity. In this study, ploidy appeared to have an effect on some of the immune parameters measured.

Ploidy had an effect on WBC and RBC counts, with triploids showing lower cell numbers compared to diploids. It is recognised that the third chromosome possessed by triploids is compensated for by increased cell size and reduced cell number and since the late 1950's, numerous studies have supported this, with reduced cell numbers continually observed in triploids [44], [45], [46], [47]. In terms of treatment effects, WBC counts were the lowest in both the ADJ groups and the triploid VACC group. Following vaccination, it would be expected that WBC numbers in the VACC groups increase over that of the controls. From vaccination to the 50°D sampling point, the water temperature was low (4.9 ± 0.2 °C) and so, it could be suggested that low temperature had suppressive effect on the WBC's of the innate immune response and the levels remain basal. In a previous study on diploid rainbow trout, similar cell volumes were recorded at the same temperature observed in this study [31]. Variation can also be seen in RBC counts between treatment groups, with similar levels previously exhibited in rainbow trout [31]. As such, with RBC's not playing a major role in the immune response, it could be suggested that the numbers observed are of normal population variation.

Ploidy had a significant effect on the activity of head kidney macrophages. Respiratory burst in triploids was significantly higher than in diploids, with the triploids consistently showing more than double their activity. This finding may also reflect the compensatory mechanism employed by triploid fish to deal with the reduced number of cells present and supported by previous studies which found increased activity in triploids compared to diploid counterparts [24], [47]. Treatment was not found to have a significant effect on respiratory burst. Given previous research which showed increased respiratory burst following vaccination or exposure to an antigen [48], [49], [50], it was expected that the respiratory burst activity of the VACC groups would exceed that of the controls. However, it could again be suggested that the low temperature experienced during this study was having a suppressive effect on the respiratory burst activity in the VACC groups. This is supported by a previous study in diploid rainbow trout which showed similar respiratory burst activity to that recorded in this study, at the same temperature.

In terms of phagocytosis, varied pattern of activity were recorded. Considering previous studies, triploids would be expected to show increased activity during phagocytosis [24], [51] but neither diploid nor triploid head kidney macrophages consistently showed increased activity. It would also be expected that Pi and Pa would be higher in the VACC group due to antigen stimulation of the immune response. In terms of triploid phagocytosis (i.e. Pi and Pa values), lower values were recorded in the VACC groups compared to the controls. It could be suggested that while vaccines stimulate the fish's immune response, the overall reduction in cell number in triploids may have had an effect on the overall number of yeast being consumed. Further research is needed to understand the compensatory mechanisms that may occur in triploid leucocytes, particularly macrophages, in relation to genome regulation and gene expression.

In addition, a greater percentage of triploid macrophages had a higher phagocytic capacity (Pc), consuming greater numbers of yeast cells (i.e. between 5 and 7) compared to diploid fish in the PBS and ADJ groups. Budiño et al. [24], also showed that the lower cell numbers observed in triploids may be compensated for by increased cellular activity, and due to their larger size, the cells have higher membrane surface and volume than diploid cells, thus increasing their ability to engulf particles.

No significant differences in ploidy were observed in complement activity (SCH50%). The results obtained concur with the findings of a 19 day study undertaken by Langston et al. [34], in which a decrease in the ACH50% was observed for both ploidies at 2 and 3 days post-injection with lipopolysaccharide. In our study, complement activity was only measured at 50°D (8 days post-injection), which suggests that the early changes observed by Langston et al. [34] may have been missed, and the results obtained reflect recovering activity levels, and further vaccination trials should be undertaken to assess ploidy differences in complement activity at earlier time-points. Complement activity was lowest in the PBS injected group, followed by ADJ group, with the VACC group exhibiting the highest activity, although not significantly different. Other studies have found increased complement activity in vaccinated fish compared to unvaccinated controls [52], [53].

In this study, antibody response was assessed at 750°D post-vaccination, and ploidy was not found to have a significant effect on the antibody response obtained. There was, however, a trend for triploids to have greater antibody response than the diploids. This is supported by the results from Kusuda et al. [51], who revealed a non-significant trend for greater agglutinating antibody titres in triploid ayu (*Plecoglossus altivelis*). It is encouraging that triploids are able to produce similar levels of antibodies to diploids. There was a significant treatment effect on antibody response within the ploidy groups, with higher antibody responses recorded in vaccinated fish compared to the non-vaccinated groups, in accordance with other studies [35], [48]. The antibody response obtained in vaccinated fish was lower than expected, from other studies examining antibody responses in fish vaccinated against furunculosis [35], [36], possibly due to the vaccination temperature used [54], [55], however this ultimately did not affect the efficacy elicited by the vaccine.

Vaccine efficacy was confirmed by comparing the level of mortalities in each ploidy/treatment group when experimentally infected with *A. salmonicida*. While much is still unknown about the effect of ploidy on disease resistance [13], numerous studies investigating this issue have demonstrated equal or greater disease resistance in triploid [3], [24], [34], [56]. This study is clearly in support of this as no significant effect of ploidy on mortality was evident.

In conclusion, this study showed that a commercial furunculosis vaccine was equally effective in protecting diploid and triploid Atlantic salmon from infection by *A. salmonicida,* and ploidy did not affect the severity of the adhesions that occurred in vaccinated fish. The immune response of the ploidies were found to differ, however, with increased respiratory burst observed in triploids. It has been suggested that the differences seen may due to a compensatory mechanism for the reduced number of cells present in triploids. Ploidy also did not have a significant effect on levels of mortalities or vaccine efficacy during an experimental infection with *A. salmonicida* post-vaccination. Overall, the findings of this study contribute to knowledge that triploid salmon appear to be as robust as diploid siblings and provides a base for further research into the immune response of triploid Atlantic salmon.

## Figures and Tables

**Table 1:** Comparison of phagocytic capacity between diploids and triploids in each treatment group.

		Active macrophages consuming a given number yeast cells (%)							
Treatment	Ploidy	1	2	3	4	5	6	7	+
PBS	Diploid	39.3^a^ ± 2.6	24.7^a^ ±2.8	16.8 ± 4.1	8.7 ± 1.5	4.9^b^ ± 1.8	2.4^b^ ± 1.2	1^b^ ± 0.7	2.2^b^ ± 1.2
	Triploid	25.7^b^ ± 4.7	19.2^b^ ± 2.7	16.0 ± 2.7	12.9 ± 3.6	10.0^a^ ± 2.0	5.4^a^ ± 0.9	5.2^a^ ± 1.6	5.6^a^ ± 1.9
ADJ	Diploid	33.5 ± 6.1	20.2 ± 1.4	18.0 ± 3.1	9.9 ± 2.8	9.0 ± 1.6	3.1 ± 1.0	3.4 ± 1.2	3.0 ± 2.0
	Triploid	35.7 ± 7.2	19.0 ± 5.9	13.6 ± 4.6	8.9 ± 1.3	6.8 ± 2.4	5.5 ± 2.2	5.0 ± 2.3	4.5 ± 2.3
VACC	Diploid	31.2 ± 7.7	15.8 ± 1.5	14.4 ± 3.0	10.2^a^ ± 1.3	8.9 ± 3.1	5.6^a^ ± 1.8	5.8^a^ ± 1.7	8.1 ± 3.5
	Triploid	46.0 ± 9.3	17.3 ± 5.7	10.6 ± 3.6	5.7^b^ ± 2.6	5.4 ± 1.8	2.3^b^ ± 1.6	2^b^ ± 1.6	10.8 ± 5.3

Significant differences between ploidy are indicated by different lowercase superscripts.
